# New α-Glucosidase Inhibitory Triterpenic Acid from Marine Macro Green Alga *Codium dwarkense* Boergs

**DOI:** 10.3390/md13074344

**Published:** 2015-07-14

**Authors:** Liaqat Ali, Abdul Latif Khan, Lubna Al-Kharusi, Javid Hussain, Ahmed Al-Harrasi

**Affiliations:** 1 UoN Chair of Oman’s Medicinal Plants and Marine Natural Products, University of Nizwa, Birkat Al-Mouz, Nizwa-616, Oman; E-Mails: latifepm78@yahoo.co.uk (A.L.K.); javidhej@unizwa.edu.om (J.H.); 2 Marine Science and Fisheries Center, Ministry of Agriculture and Fisheries Resources, Muscat-113, Oman; E-Mail: lubna.alkharusi@gmail.com; 3 Department of Biological Sciences and Chemistry, University of Nizwa, Birkat Al-Mauz, Nizwa-616, Oman

**Keywords:** *Codium dwarkense*, isolation, characterization, enzyme inhibition

## Abstract

The marine ecosystem has been a key resource for secondary metabolites with promising biological roles. In the current study, bioassay-guided phytochemical investigations were carried out to assess the presence of enzyme inhibitory chemical constituents from the methanolic extract of marine green alga—*Codium dwarkense*. The bioactive fractions were further subjected to chromatographic separations, which resulted in the isolation of a new triterpenic acid; dwarkenoic acid (**1**) and the known sterols; androst-5-en-3β-ol (**2**), stigmasta-5,25-dien-3β,7α-diol (**3**), ergosta-5,25-dien-3β-ol (**4**), 7-hydroxystigmasta-4,25-dien-3-one-7-*O*-β-d-fucopyranoside (**5**), 7-hydroxystigmasta-4,25-dien-3-one (**6**), and stigmasta-5,25-dien-3β-ol (**7**). The structure elucidation of the new compound was carried out by combined mass spectrometry and 1D (^1^H and ^13^C) and 2D (HSQC, HMBC, COSY, and NOESY) NMR spectroscopic data. The sub-fractions and pure constituents were assayed for enzymatic inhibition of alpha-glucosidase. Compound **1** showed significant inhibition at all concentrations. Compounds **2**, **3**, **5**, and **7** exhibited a dose-dependent response, whereas compounds **4**–**6** showed moderate inhibition. Utilizing such marine-derived biological resources could lead to drug discoveries related to anti-diabetics.

## 1. Introduction

Marine algae have been demonstrated as an important source of novel secondary metabolites with a broad range of biological functions [1,2]. The members of the marine green algal genus *Codium* are well known for their anticancer activities [3,4]. The genus *Codium* is comprised of more than 40 species, many of which are reported to be from the coastal areas of Arabian Sea. These Codium species are considered as important sources of sterols and oxygenated derivatives of sterols [5,6,7]. Several reports revealed that oxygenated sterols possess interesting biological activities [7,8,9]. The aqueous extracts and clerosterol isolated from *C. fragile* possess marked anticoagulant activity [10] and cytotoxicity [3], respectively. Acylic diterpenes from *C. decorticatum* showed cytoxicity [11], whereas the secondary metabolites from *C. iyengarii* have been reported to exhibit significant antibacterial activity [12]. However, in spite of the chemical and biological potential reported for the genus *Codium*, *C. dwarkense* has been frequently overlooked when elucidating for the diversity of chemical constituents.

In addition, chemical constituents from marine-derived living organisms can lead to the exploration of drugs for various human ailments. Among these, diabetes is a serious metabolic disorder, which is characterized by high blood glucose levels [13]. According to World Health Organization estimates, global prevalence of diabetes is about 9% (or 150 million people), causing 1.5 million deaths in 2012 [14]. The best known therapeutic approach is to suppress the absorption of glucose via inhibition of related enzymes, especially α-glucosidase. Inhibiting this enzyme slows the elevation of blood sugar level after carbohydrate-rich food intake [15,16]. Overall, postprandial inhibition strategies suggest the reduction of oligosaccharide hydrolyses rate in the lower part of the small intestine, which slows the rate of glucose absorption in the blood [17]. In such a scenario, exploring for α-glucosidase inhibitors is deemed essential, whereas natural products of various plant origins have been considered a vital source [13,18]. Marine-based plants provide a potent resource for finding α-glucosidase inhibitors, as previously described by other researchers [15,19].

**Figure 1:**
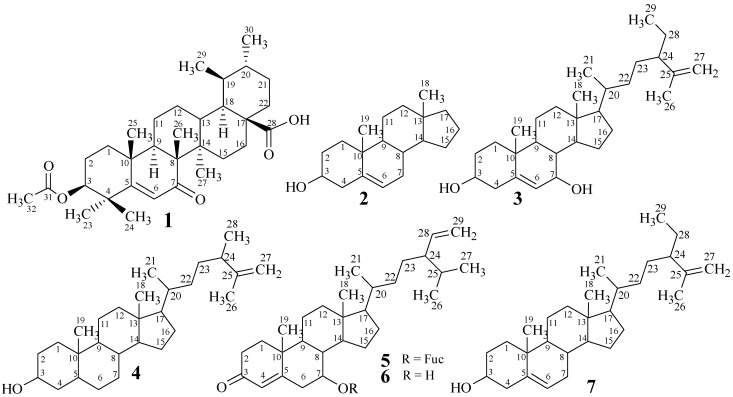
Structures of Compounds **1**–**7**.

Very little is known about the biological role of *C. dwarkense*. A previous report of Siddhanta and Shanmugam [20,21,22] linked the anti-coagulant activity to sulphated polysaccharides purified from the crude extract of this macro-alga. The present study investigates the chemical constituents of *C. dwarkense* as a part of our study on marine natural products from the Omani waters. We obtained promising results by isolating one new (**1**) and six known (**2**–**7**) secondary metabolites (Figure 1). The structures were confirmed through spectroscopic techniques. The enzyme inhibitory potential of these secondary metabolites, along with the corresponding fractions, was also carried out using an α-glucosidase enzyme inhibition assay.

## 2. Results and Discussion

### 2.1. Structure Elucidation

The algal samples of *C. dwarkense*, collected from the Gulf of Oman, were extracted in methanol (100%), and the crude methanolic extract was subjected to repeated column chromatography. As a result of the various chromatography experiments, ten different fractions were obtained at various polarity solvent systems. All the fractions were assayed for their potential effects in an α-glucosidase enzyme inhibition activity. The bioactive fractions were selected for detailed chromatographic analysis using recycling preparative High Performance Liquid Chromatography (HPLC) to obtain pure chemical constituents (**1**–**7**).

**Figure 2:**
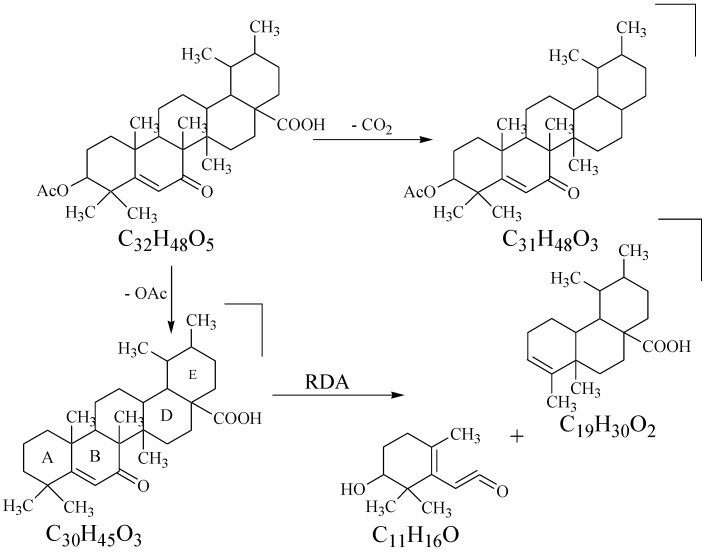
Major Mass Fragments in Compound **1**.

Compound **1** was isolated as an amorphous powder through preparative recycling HPLC. The appearance of a pink color on the TLC plates, when sprayed with cerric sulphate reagent followed by heating, was indicative of the presence of a terpenoid skeleton of the molecule. The analysis of the pseudo-molecular ion peak at *m*/*z* 513.3584 [M + H]^+^ in the High Resolution Electrospray Ionization Mass Spectrometry (HR-ESI-MS), along with the ^1^H and ^13^C NMR data, suggested the molecular formula C_32_H_48_O_5_ for compound **1**, with eight indices of hydrogen deficiency, thus supporting the pentacyclic system with one olefinic and two carbonyl groups. Further fragments appeared at *m*/*z* 468 and 453 due to a loss of CO_2_ and OAc, respectively (Figure 2). The RDA (Retro-Diels-Alder) fragmentation, which is characteristic of Δ^5^ ursene type triterpenes, was observed by the presence of a base peak at *m*/*z* 290. The IR spectrum indicated the presence of hydroxyl (3410 cm^−1^), ester and acidic carbonyls (1735 and 1688 cm^−1^, respectively), and the trisubstituted double bond (1628 and 812 cm^−1^) by the appearance of characteristic absorption bands for these groups.

The ^1^H NMR spectrum (Table 1) of **1** displayed singlets for five tertiary methyl (δ 0.81 (Me-23), 0.93 (Me-24), 1.14 (Me-26), 1.18 (Me-27), and 1.33 (Me-25)) and doublets for two secondary methyl (δ 0.78 (d, *J* = 6.4 Hz, Me-29) and 1.23 (d, *J* = 6.4 Hz, Me-30)) groups. The tertiary methyl group of the acetyl moiety (Me-32) also appeared as a singlet at δ 2.07. The presence of these methyl groups was further confirmed by ^13^C NMR spectrum, which displayed signals for tertiary methyls at δ 28.9 (Me-23), 21.1 (Me-24), 13.3 (Me-26), 18.3 (Me-27), 20.5 (Me-25), and 21.3 (Me-32), while secondary methyls appeared at δ 17.4 (Me-29) and 23.8 (Me-32). These observations were indicative of the ursane-type skeleton in the molecule. The olefinic proton (H-6) was observed at δ 5.54 as a broad singlet, whereas the proton (H-3) geminal to the acetyl group was observed at δ 5.29 (1H, br s). The ^13^C NMR spectra (BB) of compound **1** (Table 1) contained 32 signals, the multiplicities of which were determined by DEPT experiments and, thus, resolved into seven methine, eight methyl, eight methylene, and nine quaternary carbons. The signals for eight methyl groups including one acetyl methyl, coupled with a downfield signal for quaternary carbon at δ 179.1 (C-28) and a methine carbon at δ 73.1 (C-3), proposed that the compound is likely to be a derivative of ursolic acid [23]. The olefinic moiety in ring-B was further confirmed by the presence of signals for methine carbon (C-6) at δ 130.5. The downfield shift of C-5 at δ 164.9 and a signal at δ 199.4 in ^13^C NMR spectrum indicated the presence of the α*-*β unsaturated system with alkene and ketone moieties. The quaternary carbon at δ 170.2, coupled with the indication of acetyl group in ^1^H NMR spectrum also confirmed the presence of acetyl substitution in the molecule.

**Figure 3:**
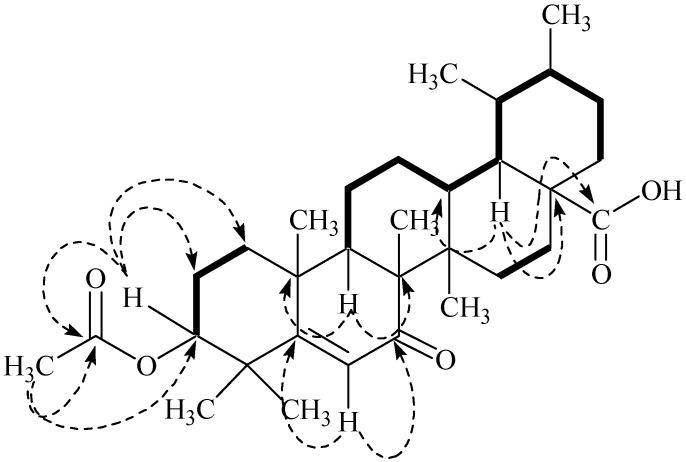
Key COSY (bold) and Selected HMBC (arrows) Correlations in Compound **1**.

The HSQC spectrum was helpful for establishing the ^1^H-^13^C connectivities whereas the long-range ^1^H-^13^C correlations (HMBC; Figure 3) were used to assign various substituents in the molecule to combine different sub-structures in **1**. The proton H-3, which is geminal to the acetyl group showed long range heteronuclear correlations with C-1 (δ 40.9), C-2 (δ 23.5), and C=O (δ 170.2); H-6 proton showed interactions with C-5 (δ 164.9), C-6 (δ 130.5), and C=O (δ 199.4); H-9 proton showed cross peaks with C-8 (δ 45.1) and C-10 (δ 37.3); and H-18 showed cross peaks with C-13 (δ 50.4), C-17 (δ 43.8), and C=O (δ 179.1), thus indicating the relative positions of these groups in the molecule.

**Table 1:** ^13^C and ^1^H NMR data (150 and 600 MHz; CDCl_3_) and HMBC correlations for compound **1**.

C. No.	^13^C (δ)	^1^H (δ)	Multiplicity	Key HMBC
1	40.9	1.50, m	CH_2_	
2	23.5	2.11, m; 2.21, m	CH_2_	
3	73.1	5.29, br s	CH	1,2,32
4	33.9	-	C	
5	164.9	-	C	
6	130.5	5.54, s	CH	5,7
7	199.4	-	C	
8	45.1	-	C	
9	60.1	2.39, m	CH	8,10
10	37.3	-	C	
11	27.2	1.87, m; 1.98, m	CH_2_	
12	29.7	1.89, m; 2.08, m	CH_2_	
13	50.4	1.38, m	CH	
14	46.3	-	C	
15	32.8	1.45, m; 1.66, m	CH_2_	
16	34.6	2.29, m; 2.51, m	CH_2_	
17	43.8	-	C	
18	59.0	1.54, m	CH	13,17,28
19	39.2	1.37, m	CH	
20	39.3	0.92, m	CH	
21	30.9	1.27, m; 1.42, m	CH_2_	
22	27.5	0.99, m, 1.20, m	CH_2_	
23	28.9	0.81, s	CH_3_	4
24	21.1	0.93, s	CH_3_	4
25	20.5	1.33, s	CH_3_	
26	13.3	1.14, s	CH_3_	
27	18.3	1.18, s	CH_3_	
28	179.1	-	C	
29	17.4	0.78, d, *J* = 6.5 Hz	CH_3_	
30	23.8	1.23, d, *J* = 6.5 Hz	CH_3_	
31	170.2	-	COCH_3_	
32	21.3	2.07, s	COCH_3_	3,31

**Figure 4:**
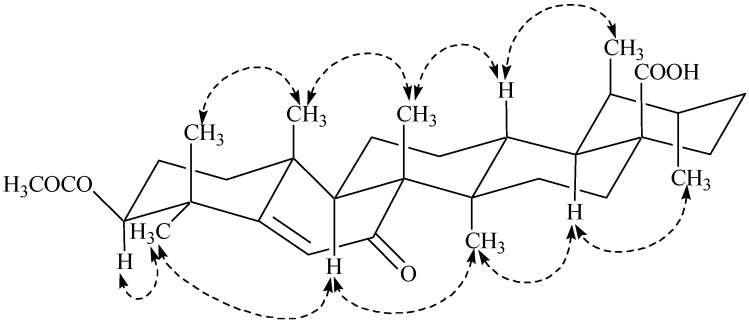
Key NOESY Interactions in Compound **1**.

**Figure 5:**
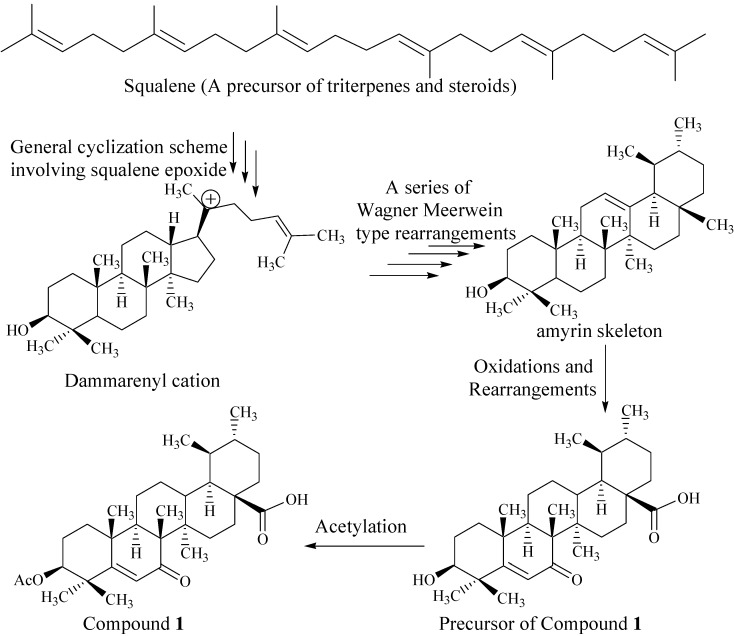
Biogenetic pathway for compound **1**.

The relative orientation of various groups in **1** was assigned by the analysis of NOESY correlations (Figure 4). The NOESY cross peaks between Me-26 and Me-25 indicated them to be axial and on the same face of the molecule, while the absence of correlation between H-9 and Me-26 suggested H-9 to be opposite to Me-26 and Me-25. Thus, the *trans* ring-junction at C-8/C-9 was confirmed with the axial orientations of Me-26 and H-9 (Figure 4). Furthermore, coupled with the biogenetic consideration (Figure 5), the NOESY correlation between Me-24, Me-25, Me-26, H-13, and Me-29 indicated them to be on the same face of the molecule with a β-axial orientation. H-3, H-9, and H-18 were located on the opposite face of Me-24 with α-axial orientation, based on the absence of NOESY correlation, whereas the correlation between H-3, Me-23, H-9, Me-27, H-18, and Me-30 indicated them to be located on the same face of the molecule. Thus, β-equatorial orientation of the acetyl group at C-3 (3-OAc) relative to α-axial orientation of H-3 was also confirmed. The above evidence established the structure of **1** as 3-*O*-acetyl-7-oxo-urs-5-en-28-oic acid, which was a new constituent from *Codium dwarkense*, and was named as dwarkenoic acid (**1**), after the producing organism. The known compounds **2**–**7** were identified by the comparison of NMR data with those reported in literature [5,6,7,8,9].

#### Biogenesis of Compound **1**

Compound **1** possesses the ursane skeleton belonging to the pentacyclic triterpene system. The pentacyclic triterpene system arises from squalene [24], which is a basic precursor for most of steroids and terpenoids.

The folding of squalene oxide onto a cyclase enzyme in a chair-chair-chair-boat conformation [25] produces the dammarenyl cation as the transient product. This cation undergoes further carbocation-promoted cyclizations to form lupenyl cation with a pentacyclic ring system. Ring expansion in the lupenyl cation gives rise to the oleanyl system, which, on further hydride migrations and loss of proton, converted into the amyrin skeleton (Figure 5). Compound **1** possesses the similar skeleton to that of amyrin. It may be a biosynthetic product of esterification of a precursor for **1** (Figure 5), which is the oxidation/rearrangement product of α-amyrin at C(6) and C(28) [25].

### 2.2. Biological Evaluations

**Figure 6:**
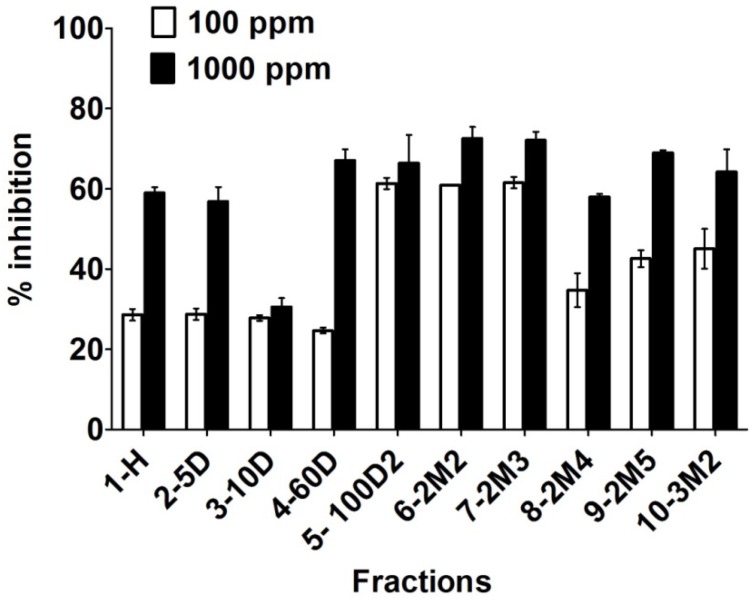
Effects of two different concentrations of various polarity fractions obtained from the crude methanolic extract of *Codium dwarkense* against α-glucosidase inhibition. The bars in the graph shows mean of three replicates with standard error.

Various fractions of crude methanolic extract of *Codium dwarkense* were subjected to enzyme inhibition assay to evaluate the potential function in suppressing α-glucosidase enzyme. The initial screening results showed significantly higher enzyme inhibition with the increasing gradient of polarity of the solvents used for fractionation (Figure 6). The enzyme inhibition increased significantly with increase in the concentration of dichloromethane (DCM) and methanol (MeOH). It was also observed that at maximum concentration (1000 μg/mL), the response against enzyme activity was significantly higher as compared to minimum concentration (100 μg/mL). Since the polar fractions resulted in significantly higher enzyme suppression level, these fractions (5-100D2, 6-2M2, and 7-2M3) were further subjected to chromatography to isolate α-glucosidase inhibitory constituents.

**Figure 7:**
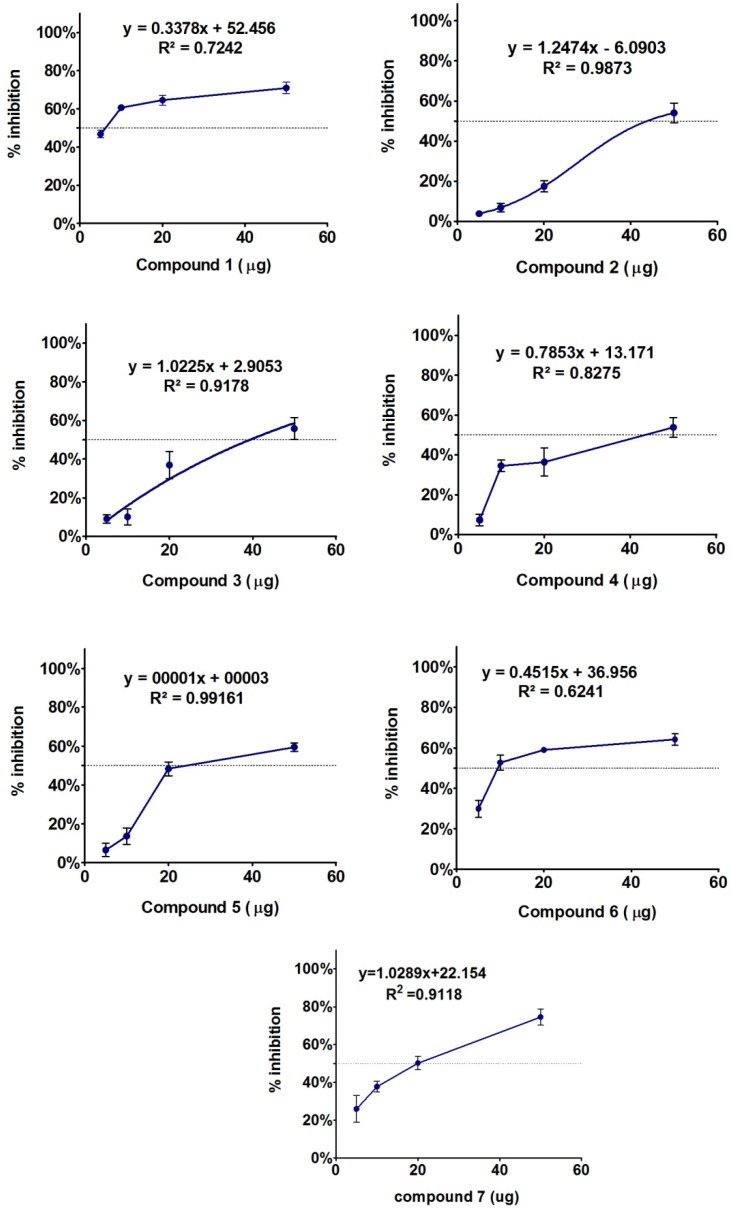
α-Glucosidase enzyme inhibitory effect of compounds **1**–**7** isolated from fractions showing higher suppression level. The graph shows mean of three replications with standard error. The inhibition absorbance was analyzed through Graphpad Prism (ver 5.05, GraphPad Software, Inc., La Jolla, CA, USA) using non-linear regression curve fit for inhibitor *vs.* effect response. The line shows the IC_50_ log curve as per dosage of the compounds. Curve values with precision near *R^2^* = 1.0 has been regarded ideal dosage with regards to standard curve.

Compound **1**–**7** were isolated and characterized through advanced chromatographic and spectroscopic techniques. These isolated compounds were subjected again to assess their potential in α-glucosidase inhibition. The compounds were assayed using three different concentrations, while the inhibition response against each dose was analyzed by non-linear regression and presented in inhibitor *vs.* response curve-fit graph (GraphPad prism 5.05, GraphPad Software, Inc., La Jolla, CA, USA). The inhibition pattern is given in detail in Figure 7. Dose-dependent enzyme inhibition non-linear curve fitting analysis showed that compound **2**, **3**, **5** and **7** are exhibiting a dose-dependent response with *R*^2^ values ranging well above 90% (Figure 7). The logIC_50_ values of Compound **1**–**7** were 3.47 ± 0.027, 5.32 ± 0.09, 3.9 ± 0.09, 9.09 ± 0.016, 16.89 ± 0.1, 20.12 ± 0.21, and 3.31 ± 0.011, respectively. However, looking at percentage of inhibition, most of the concentrations of compound **1** and **7** exhibited significantly higher activity. Compounds **2**–**5** showed comparatively moderate inhibition patterns against α-glucosidase activity (Figure 7). Previously, Siddhanta and co-workers demonstrated that the anticoagulant activity of *C. dwarkense* was linked to carbohydrate, sulphate, protein, uronic acid, and arabinose sugar content [20,21,22]. Although we did not find any α-glucosidase inhibition activity for these secondary metabolites, previously, other kinds of sterols have been reported to possess less to moderate α-glucosidase enzyme inhibition activities, as reported in literature [26,27].

## 3. Conclusions

*Codium dwarkense* is not known for its phytochemical investigations. Herein, the first comprehensive study was carried out on the isolation of biologically active secondary metabolites from *Codium dwarkense*. The identification of new sources of natural products is an important step in drug discovery. In this sense, the results from our initial study highlight the potential of *C. dwarkense* from Omani waters as a source of interesting bioactive compounds. The bioassay-guided isolation approach confirmed the identity of compounds responsible for the enzyme inhibitory potential of the crude extract. Subsequent biological evaluation of the isolated secondary metabolites indicated that compound **1** showed a higher inhibition at all concentrations. However, compound **2**, **3**, **5** and **7** showed a dose-dependent response, while compounds **4** to **6** were found to be moderately active against the tested enzyme. The results of the present study may lead to the conclusion that marine seaweeds are to be considered as a potential source for novel bioactive products, and thus play an important role in the search for natural compounds as an alternative source for the production of therapeutic agents and bioactive metabolites that are not easily synthesized and have a high activity against pathogenic microorganisms.

## 4. Experimental Section

### 4.1. General

Optical rotations were measured on a KRUSS P P3000 polarimeter (A. Kruss Optronics, Hamburg, Germany). Melting point was measured on a Thermo Scientific manual melting point apparatus. IR spectra were recorded on a Bruker, ATR-Tensor 37 spectrophotometer. EI mass spectra were recorded on mass spectrometers JEOL JMS HX 110. ESI mass spectra were recorded on QSTAR XL (Applied Biosystem, Foster, CA, USA). The capillary voltage was maintained between 5 and 5.5 kV. The ^1^H and ^13^C NMR spectra were recorded on Bruker NMR spectrometers operating at 600 MHz (150 MHz for ^13^C). The chemical shifts values are reported in ppm (δ) units and the coupling constants (*J*) are given in Hz. Data are reported as follows: Chemical shift (multiplicity (singlet (s), doublet (d), triplet (t), quartet (q) and multiplet (m)), coupling constants (Hz), and integration). Minor compounds were purified by using recycling preparative High Performance Liquid Chromatography (HPLC) by JAI using 1H/2H silica gel column with chloroform as the eluting systems. For TLC, pre-coated aluminum sheets (silica gel 60F-254, E. Merck) were used. Visualizations of the TLC plates were achieved under UV light at 254 and 366 nm, and also by spraying with ceric sulphate and ninhydrin reagents. The solvent system of 1%–5% MeOH/DCM was used to get suitable *Rf* values for the pure constituents in the TLC plates.

### 4.2. Plant Material

*Codium dwarkense* Boergs. (Codeaceae) was collected in March/April 2014 from the coastal areas of the Gulf of Oman near Sur (22°34ʹ0ʺ N; 59°31ʹ44ʺ E), in the northeastern parts of Oman. The algal specimen was then identified by Dr. Lubna Hamoud Al-Kharusi, Marine Science and Fisheries Center, Ministry of Agriculture and Fisheries Resources, Sultanate of Oman.

### 4.3. Extraction and Isolation

The algal materials were thoroughly cleaned, washed with tap water to remove the sea salts, and then freeze-dried. The freeze-dried and powdered material (*C. dwarkense*, 500 g) was defatted with *n*-hexane and then extracted with methanol at room temperature. The filtrate was evaporated in vacuum to yield 55 g of the residue. The residue was then subjected to vacuum liquid chromatography (VLC) on the basis of increasing the polarity of the organic solvents (*n*-hexane, *n*-hexane/dichloromethane, and dichloromethane/methanol) to get ten sub-fractions (Figure 6). The bioactive polar sub-fractions obtained at 100% DCM and 2% MeOH/DCM were further subjected to repeated column chromatography and the final purification was done by recycling preparative HPLC. By using the 1H/2H column in JAI recycling HPLC with 3.5 mL·min^−1^ flow rate of chloroform, compound **3** (2.4 mg), **4** (1.7 mg), **6** (1.9 mg), and **7** (2.1 mg) were purified at retention times between 40 and 50 min. along with some semi-pure sub-fractions. These semi-pure sub-fractions were subjected again to the same HPLC conditions and after two recycles compound **1** (2.3 mg) and **2** (3.9 mg) were eluted at retention time of 32 min and 41 min, respectively. Compound **5** (1.5 mg) was purified through preparative TLC plates at 1% MeOH/DCM.

#### Dwarkenoic Acid (**1**)

Amorphous powder; mp: 274–275 °C; [α]_D_^27^ +14 (*c* 0.005, CHCl_3_); IR (KBr): *ν*_max_ 3410, 1735, 1688, 1628, and 812. ^1^H NMR (600 MHz, CDCl_3_): See Table 1; ^13^C NMR (CDCl_3_, 150 MHz): See Table 1; HR-ESI-MS: *m*/*z* 513.3584 [M + H]^+^.

### 4.4. Enzyme Inhibition Assay

Enzyme inhibition assay (α-glucosidase, E.C.3.2.1.20) was performed according to the modified method of Oki *et al.* [28]. The inhibition was measured spectrophotometrically on ELISA (xMark, Bio-Rad, Hercules, CA, USA) at pH 6.9 and at 37 °C using 0.7 mM *p*-nitrophenyl α-d-glucopyranoside (PNP-G) as a substrate and 200 m units/mL enzyme, in 50 mM sodium phosphate buffer containing 100 mM NaCl. Acarbose was used as a positive control. The increment in absorption at 400 nm due to the hydrolysis of PNP-G by α-glucosidase was continuously monitored with ELISA.

## Supplementary Information

NMR and MS data for the new compound is included. This material is available free of charge via MDPI website.
