# Supplementary material for: New α-Glucosidase Inhibitory Triterpenic Acid from Marine Macro Green Alga Codium dwarkense Boergs
Source: Mar Drugs. 2015 Jul 14;13(7):4344–56. doi: 10.3390/md13074344 (PMC4515621; doi:10.3390/md13074344)
Supplement: Supplementary file 1 [file marinedrugs-13-04344-s001.doc]

**Supplementary Information**

**S1:** Structure of Compound **1**

**S2:** ^1^H-NMR (600 MHz, CD_3_OD) Spectrum of Compound **1**

**S3:** Expansion (0.7 to 2.6 ppm) of ^1^H-NMR Spectrum of Compound **1**

**S4:** ^13^C-NMR (150 MHz, CD_3_OD) Spectrum (BB) of Compound **1**

**S5:** Expansion (10 to 75 ppm) of ^13^C-NMR Spectrum (BB) of Compound **1**

**S6:** ^13^C-NMR (150 MHz, CD_3_OD) Spectrum (DEPT 135) of Compound **1**

**S7:** Expansion (12 to 53 ppm) of ^13^C-NMR Spectrum (135) of Compound **1**

**S8:** ^13^C-NMR (150 MHz, CD_3_OD) Spectrum (DEPT 90) of Compound **1**

**S9:** HSQC (600 MHz) Spectrum of Compound **1**

**S10:** Expansion (0.7 to 2.1 ppm) of HSQC Spectrum of Compound **1**

**S11:** HMBC (600 MHz) Spectrum of Compound **1**

**S12:** Expansion (0.6 to 2.5ppm) of HMBC Spectrum of Compound **1**

**S13:** Mass Spectrum of Compound **1**

**Figure S1.** Structure of Compound **1**.


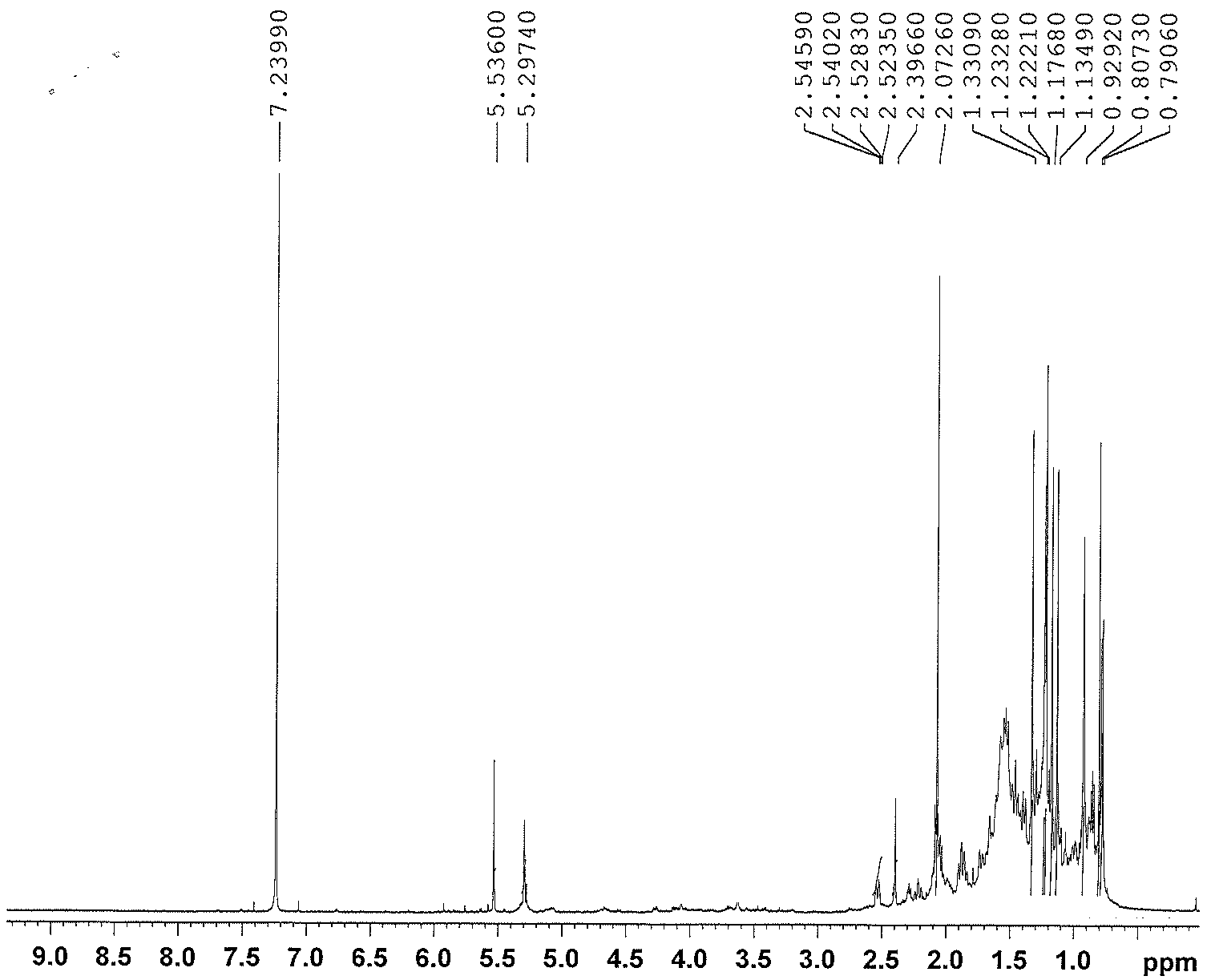


**Figure S2.** ^1^H-NMR (600 MHz, CDCl_3_) Spectrum of Compound **1**.


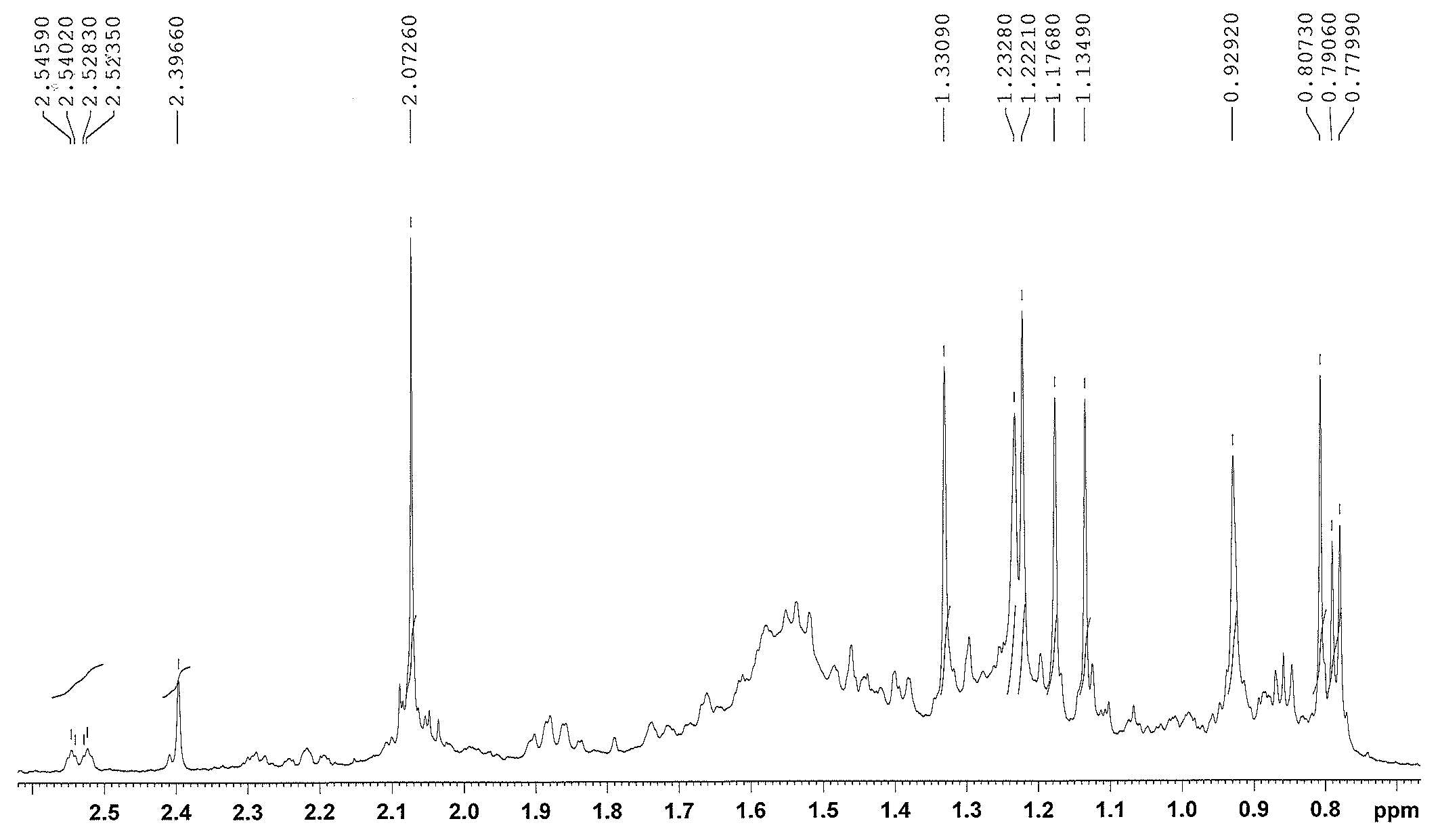


**Figure S3.** Expansion (0.7 to 2.6 ppm) of ^1^H-NMR Spectrum of Compound **1**.


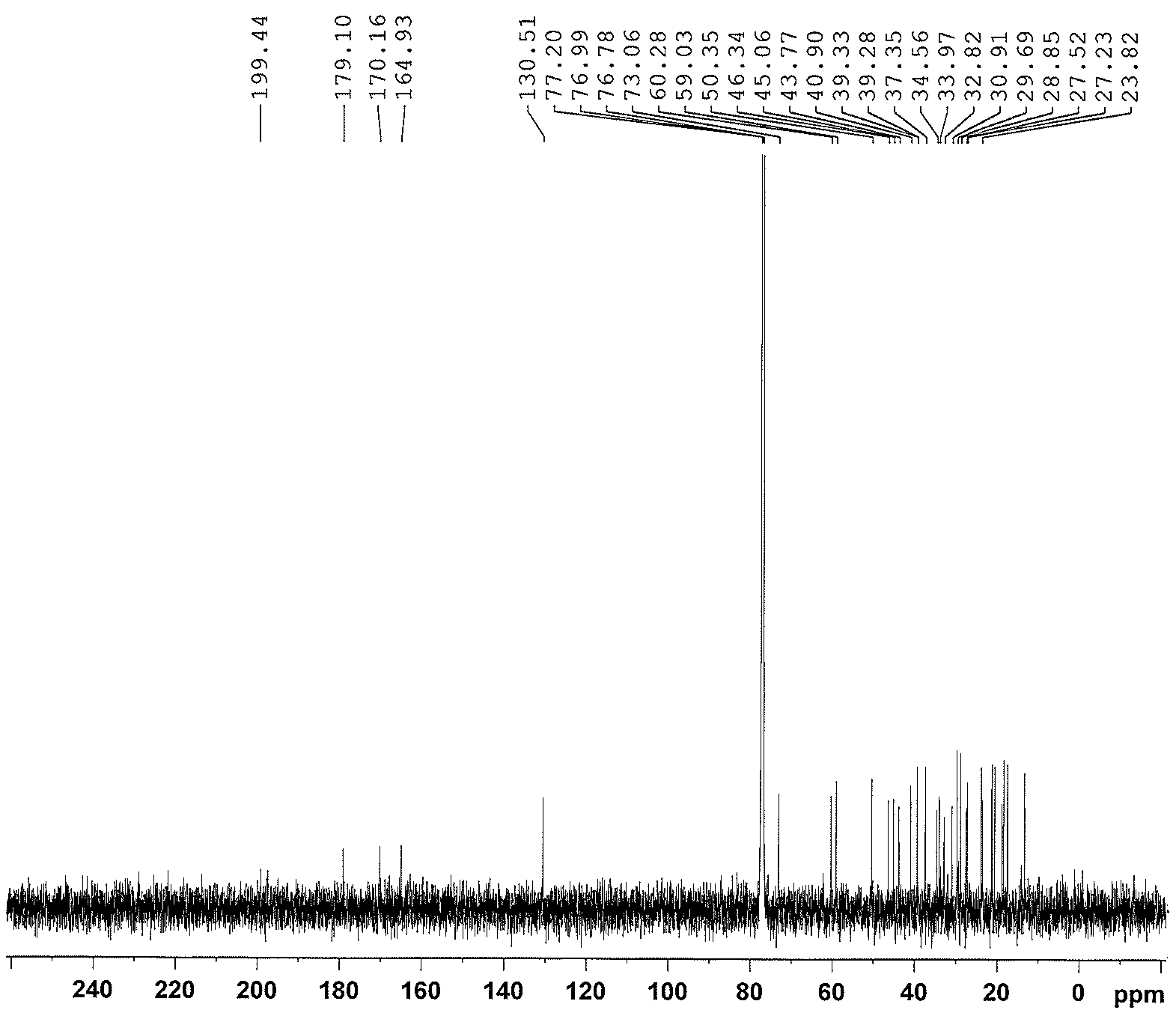


**Figure S4.** ^13^C-NMR (150 MHz, CD_3_OD) Spectrum (BB) of Compound **1**.

**
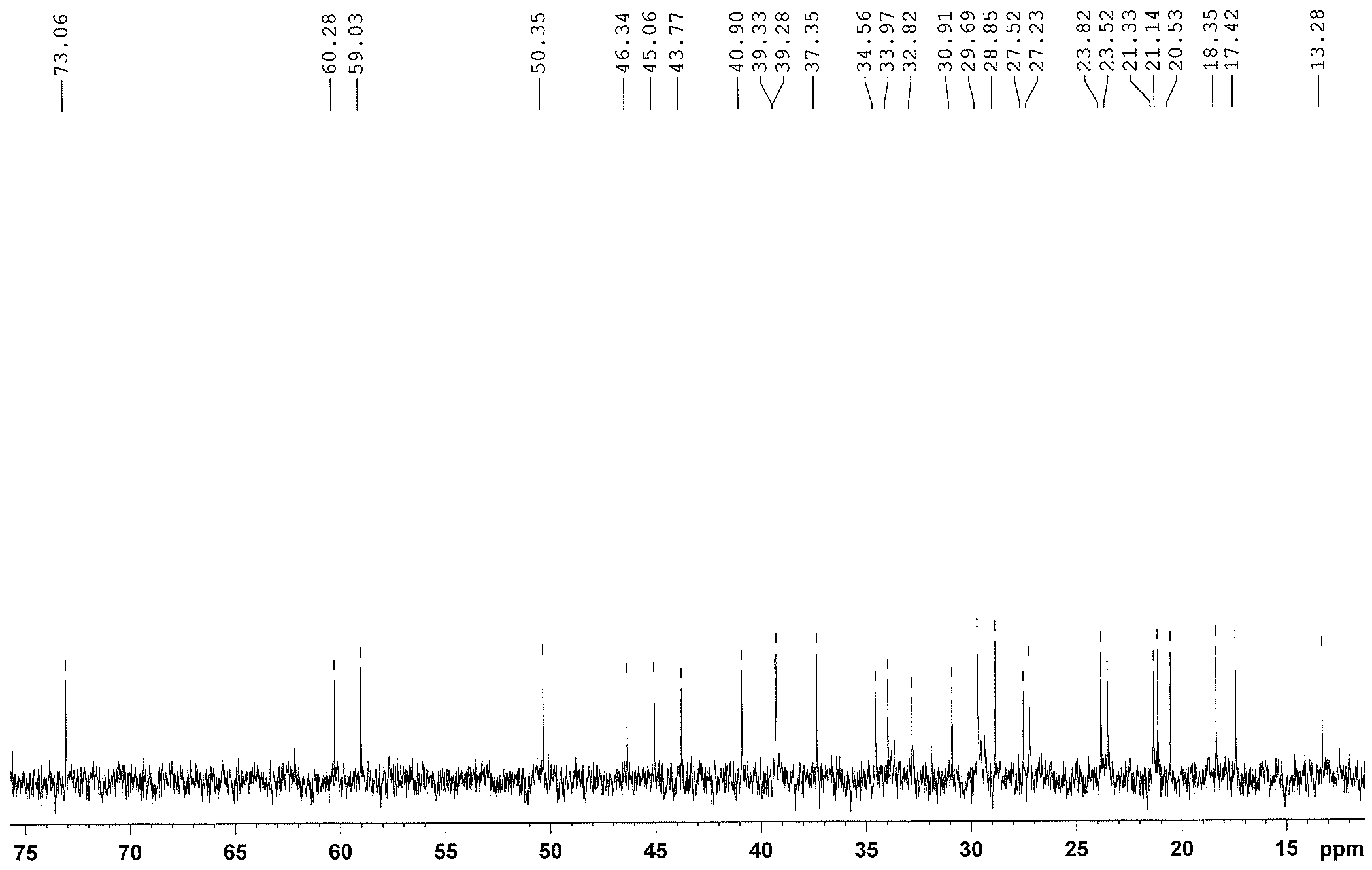
**

**Figure S5.** Expansion (10 to 75 ppm) of ^13^C-NMR Spectrum (BB) of Compound **1**.


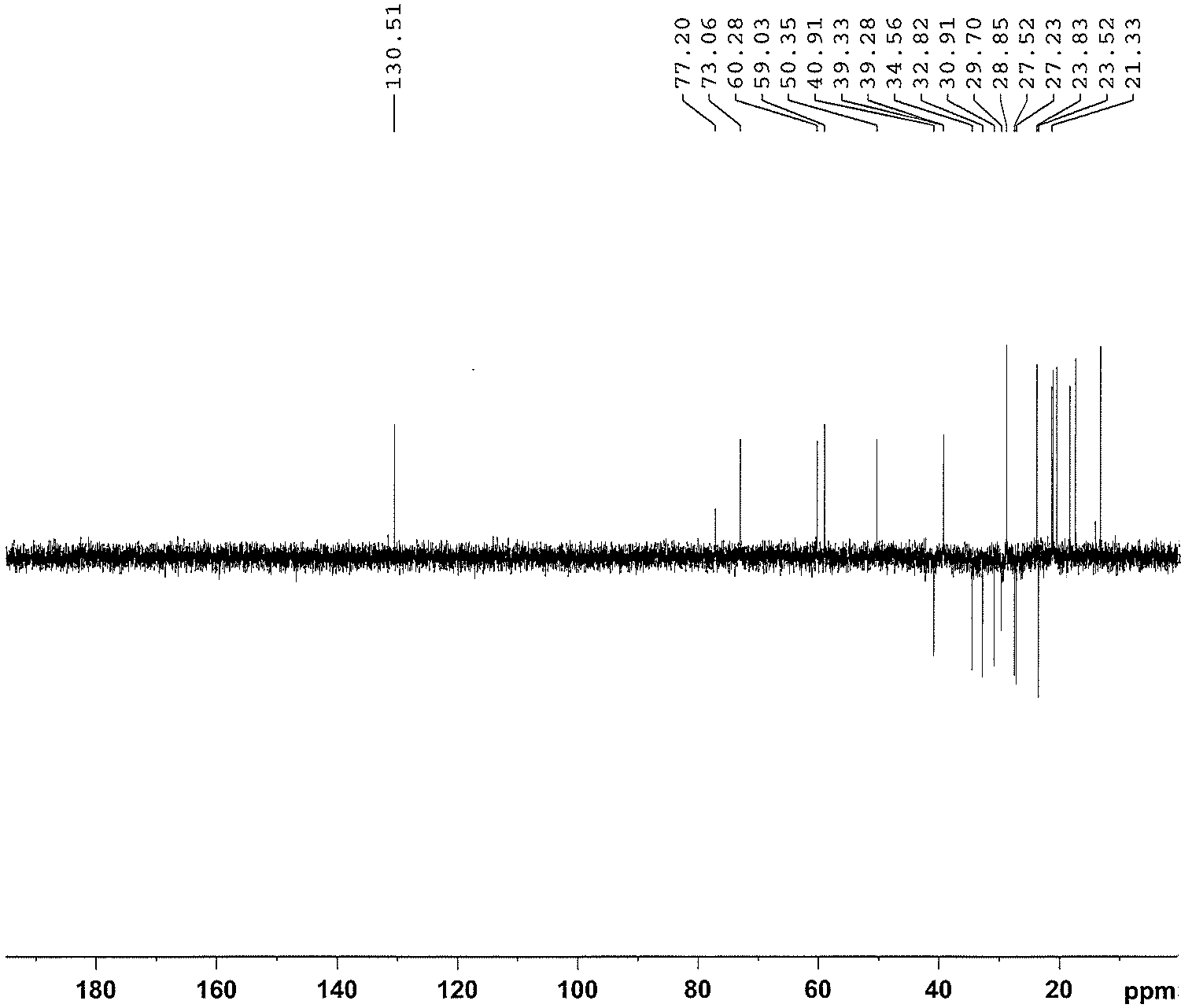


**Figure S6.** ^13^C-NMR (150 MHz, CD_3_OD) Spectrum (DEPT 135) of Compound **1**.

**
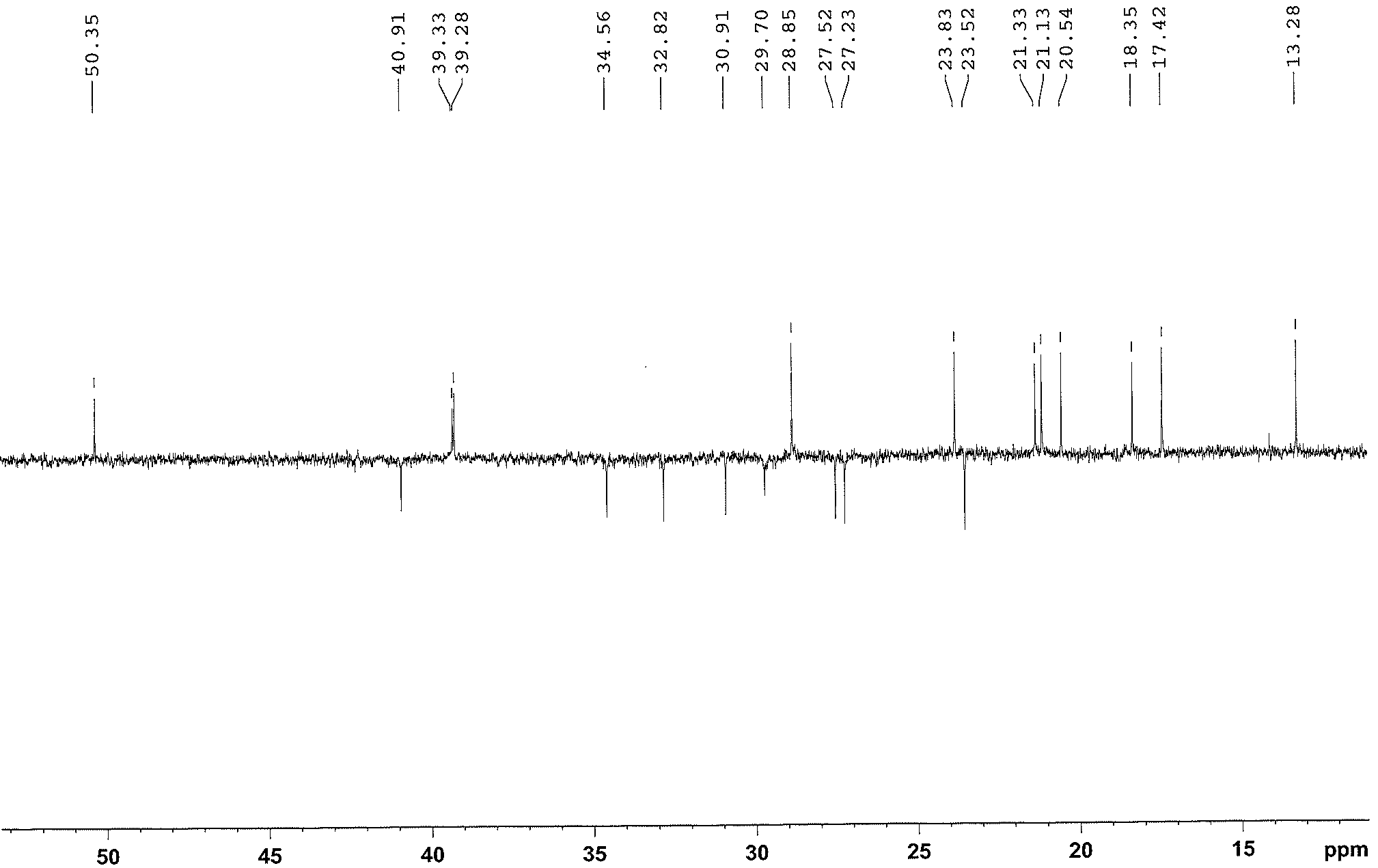
**

**Figure S7.** Expansion (12 to 53 ppm) of ^13^C-NMR Spectrum (DEPT 135) of Compound **1**.


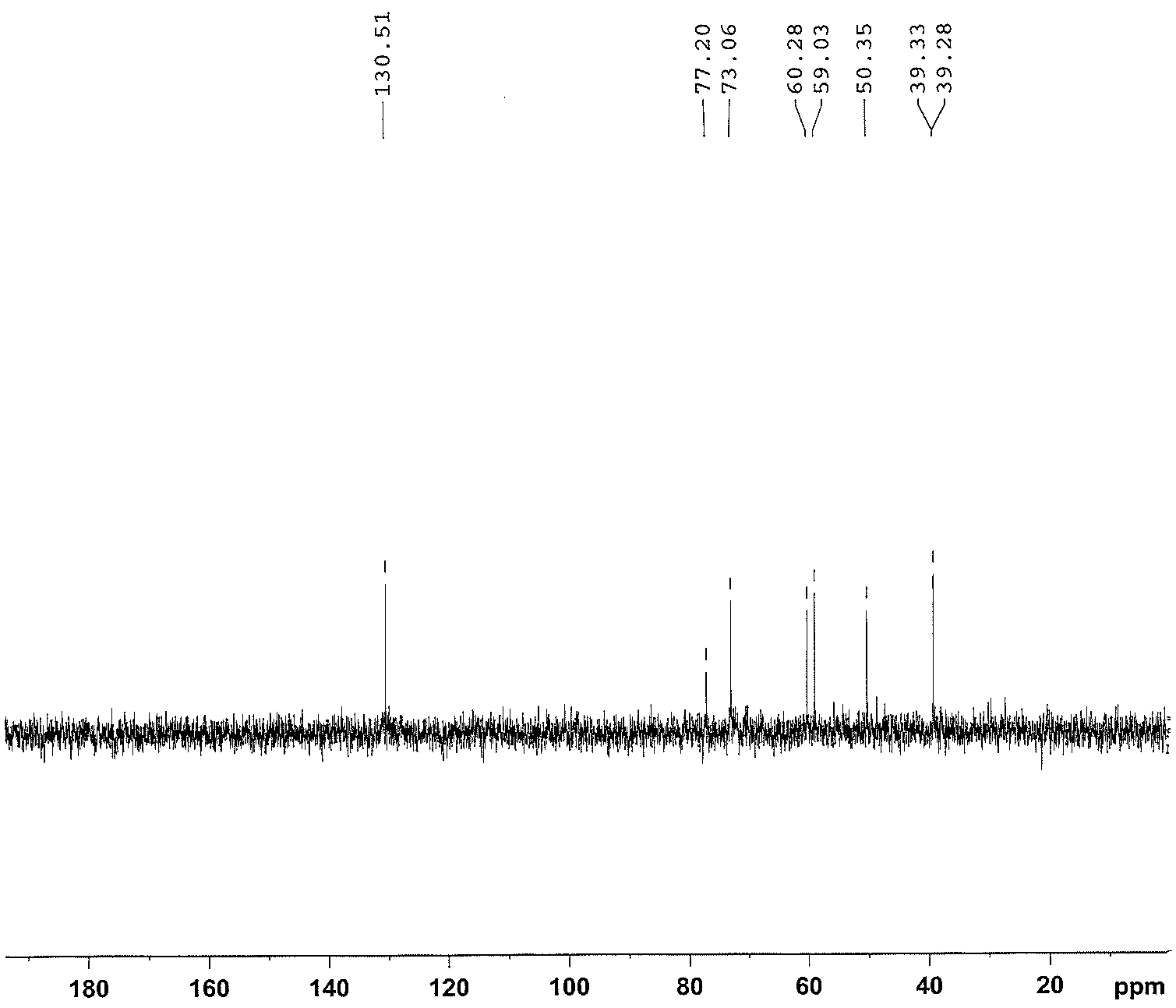


**Figure S8.** ^13^C-NMR (150 MHz, CD_3_OD) Spectrum (DEPT 90) of Compound **1**.


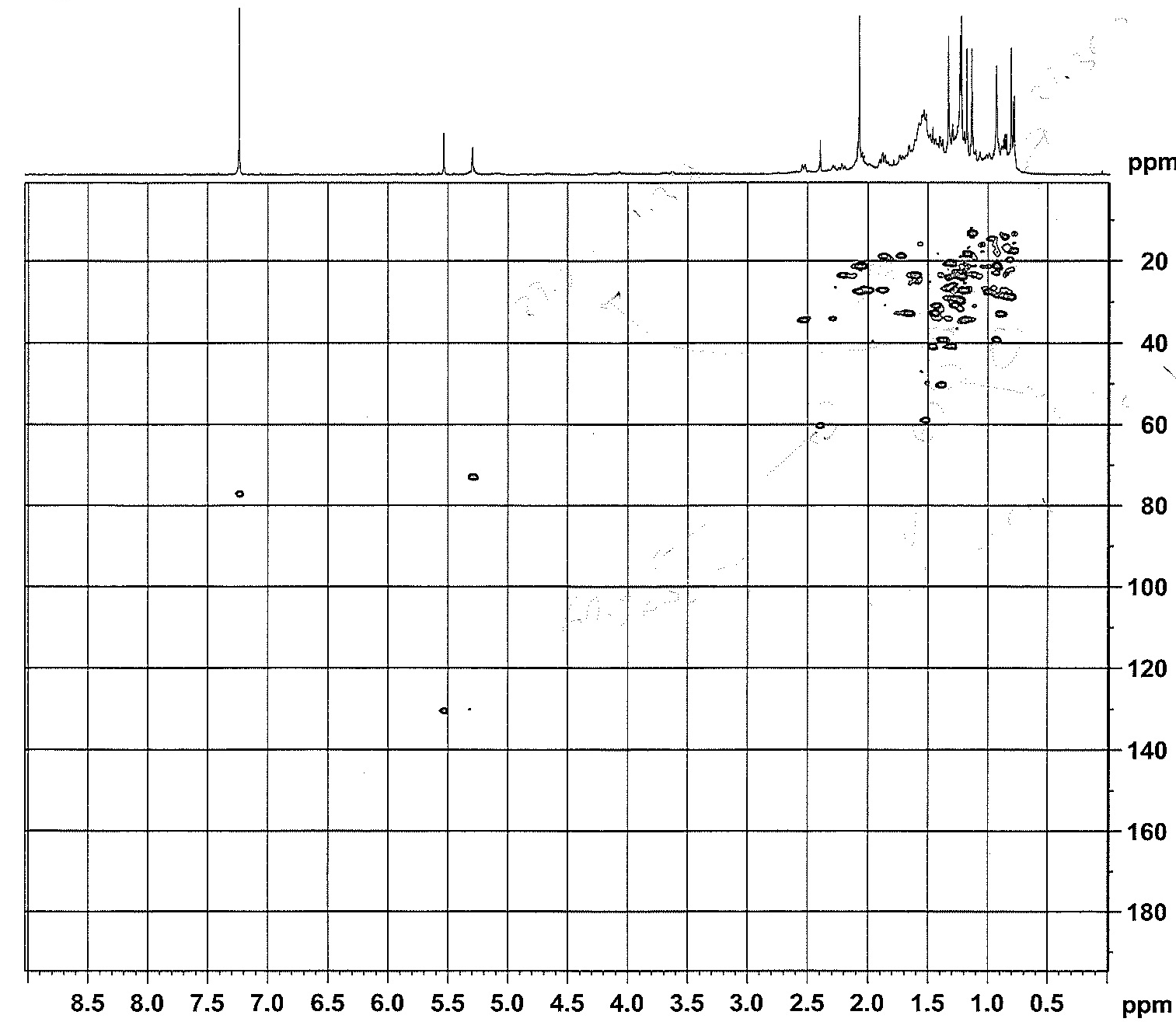


**Figure S9.** HSQC (600 MHz) Spectrum of Compound **1**.

**
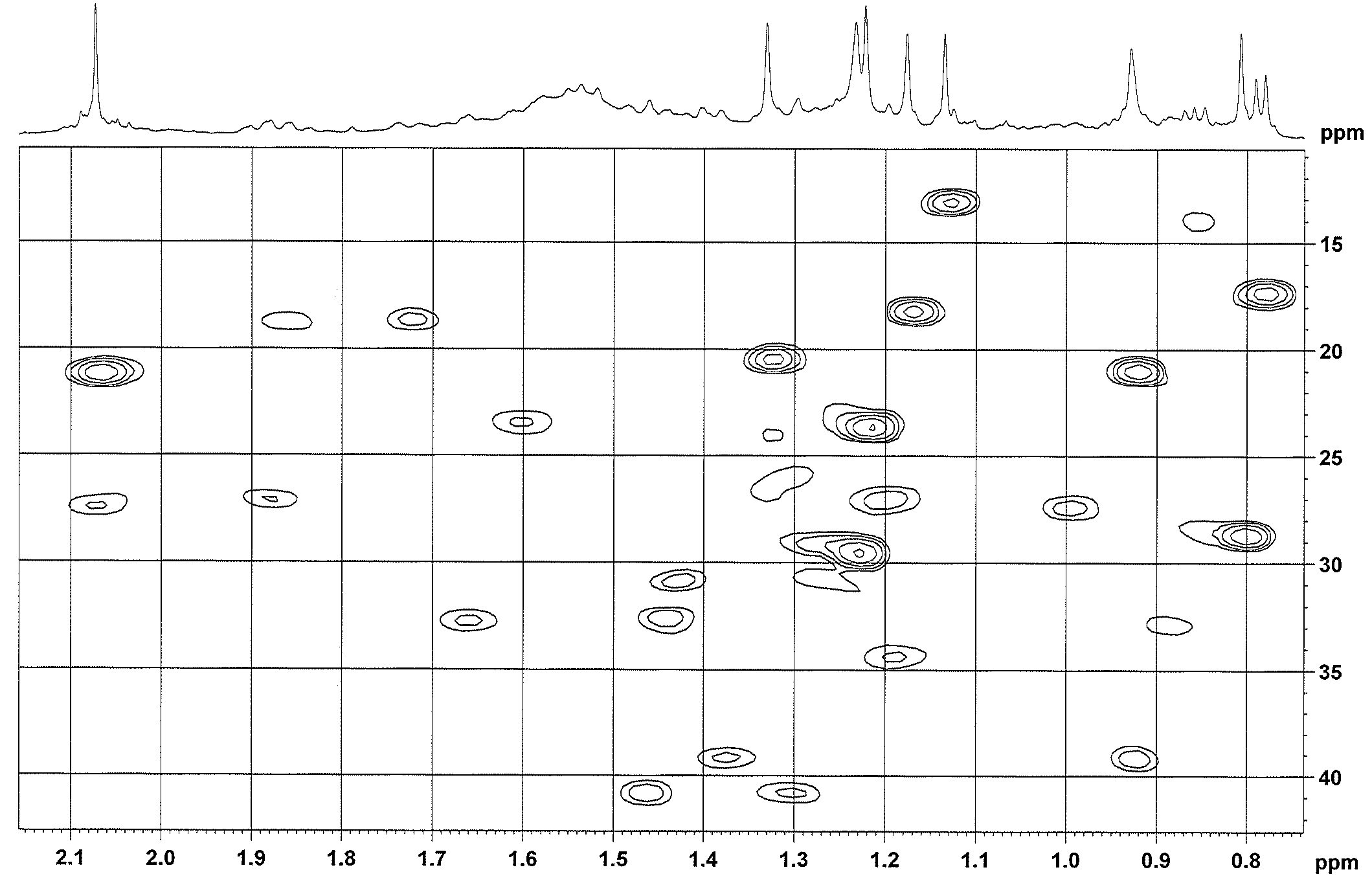
**

**Figure S10.** Expansion (0.7 to 2.1 ppm) of HSQC Spectrum of Compound **1**.


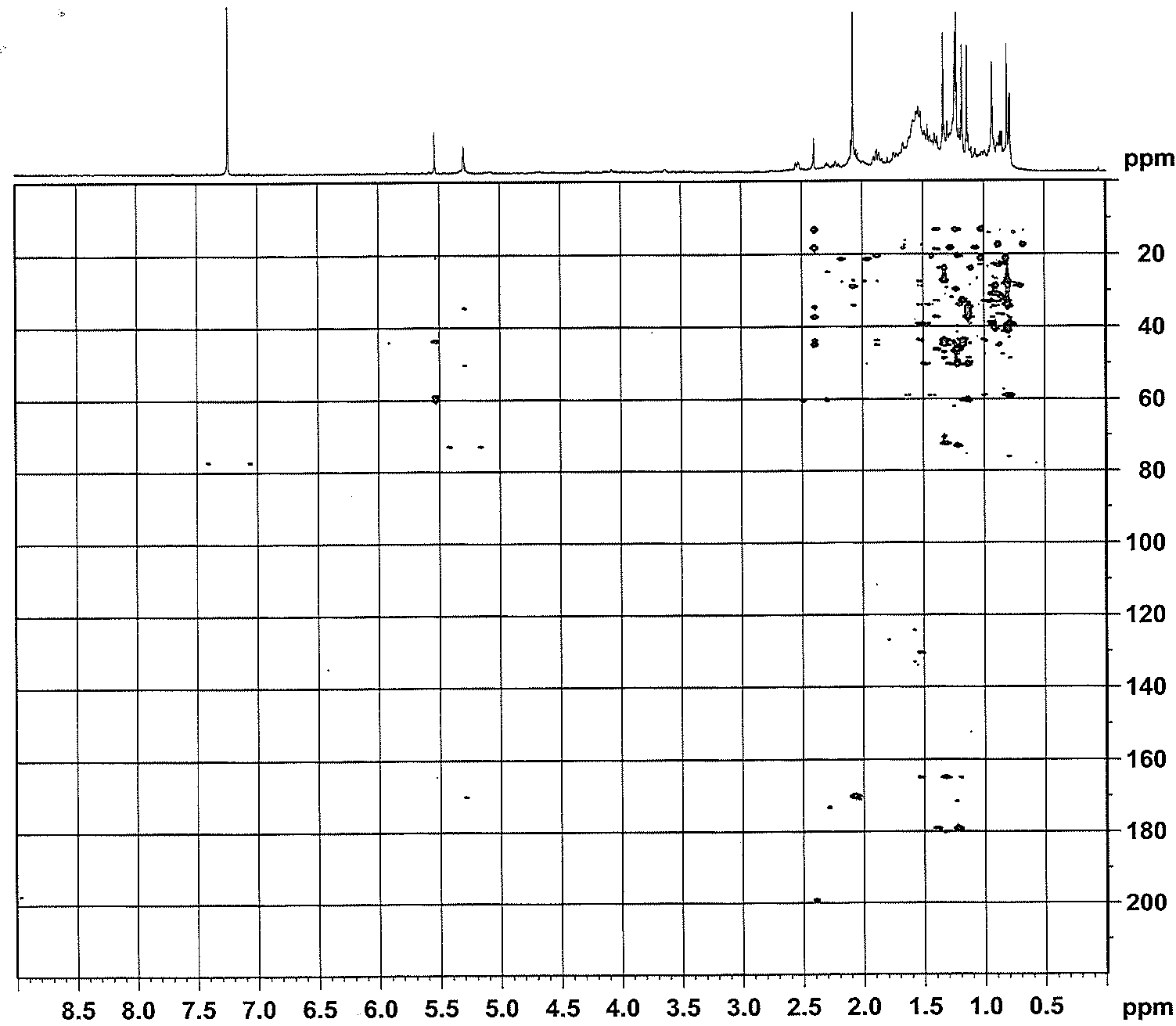


**Figure S11.** HMBC (600 MHz) Spectrum of Compound **1**.

**
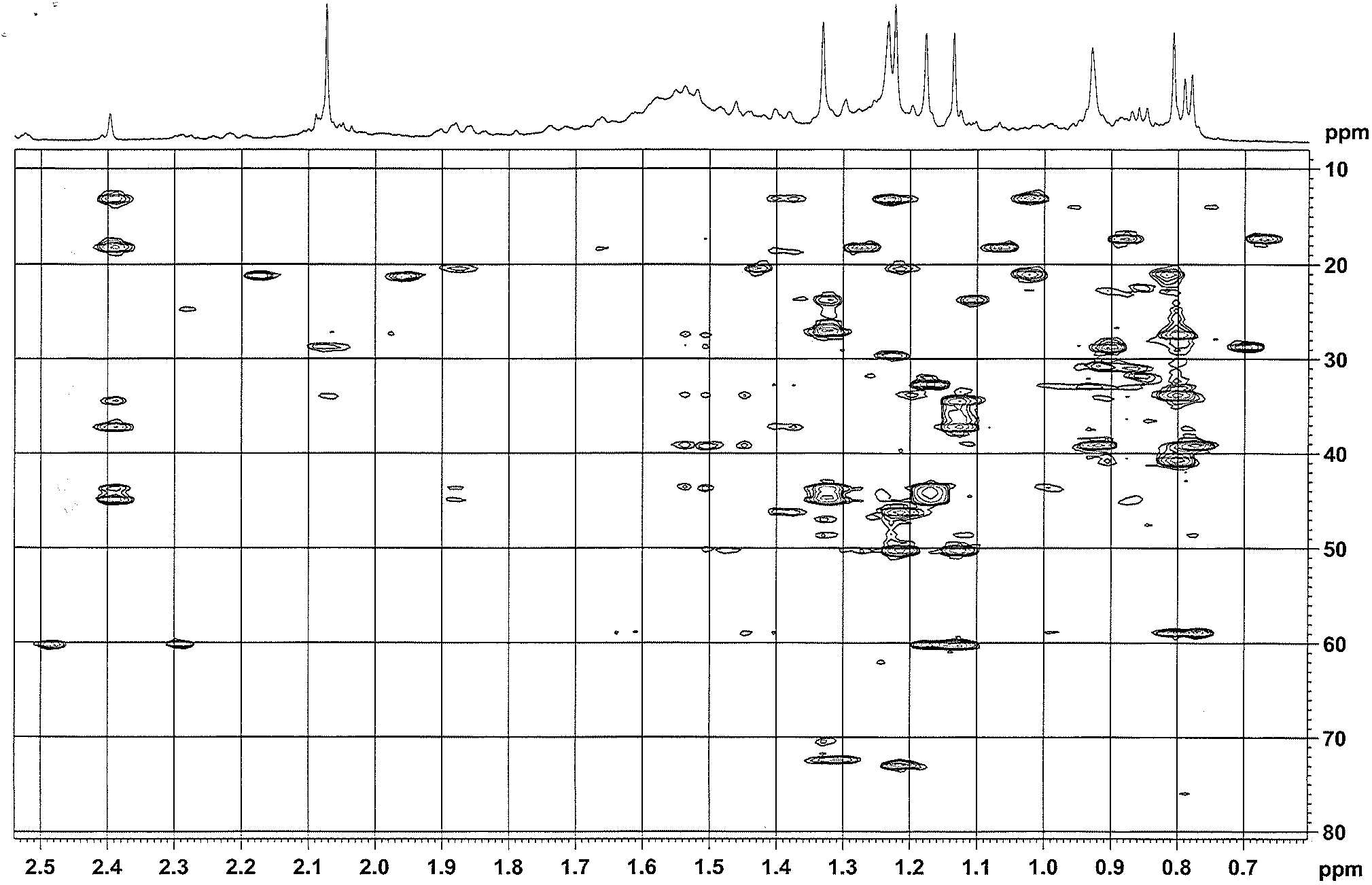
**

**Figure S12.** Expansion (0.6 to 2.5 ppm) of HMBC Spectrum of Compound **1**.


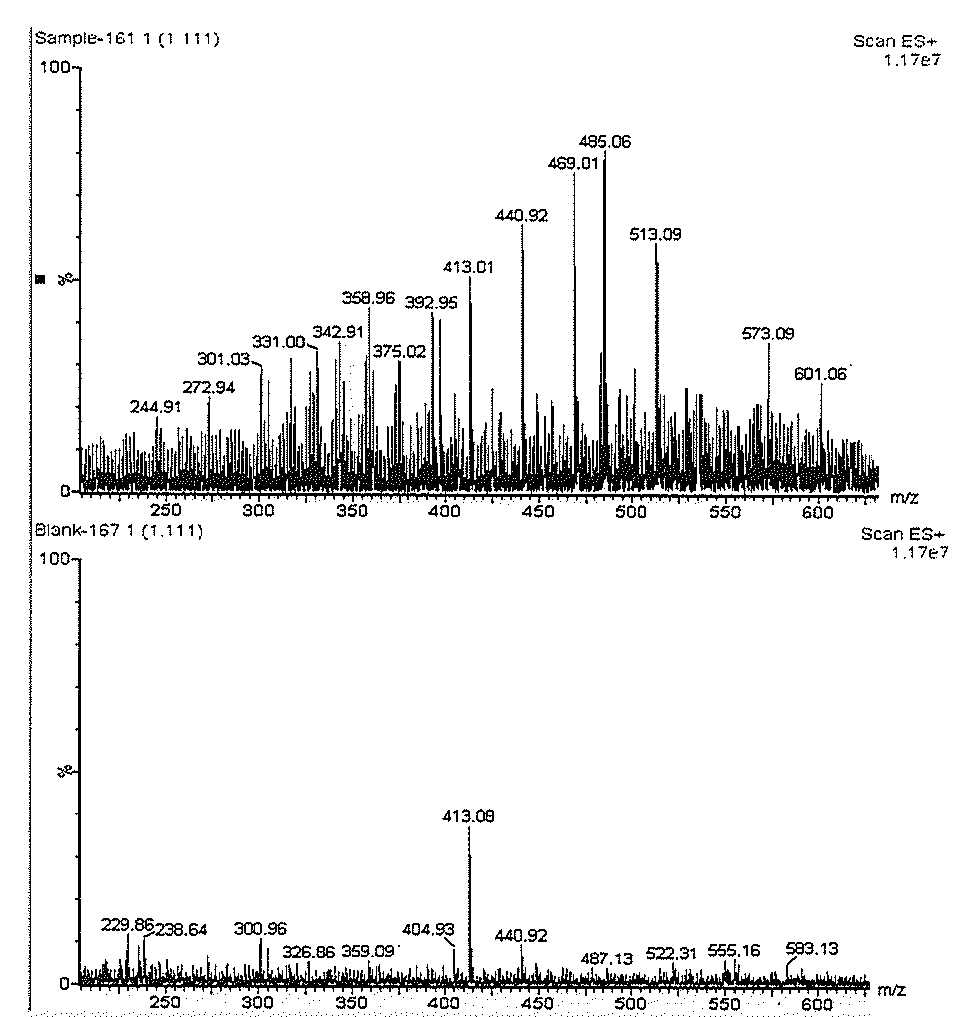


**Figure S13.** Mass Spectrum of Compound **1.**

© 2015 by the authors; licensee MDPI, Basel, Switzerland. This article is an open access article distributed under the terms and conditions of the Creative Commons Attribution license (http://creativecommons.org/licenses/by/4.0/).
